# Male sexual and reproductive health in multiple sclerosis: a scoping review

**DOI:** 10.1007/s00415-024-12250-2

**Published:** 2024-02-28

**Authors:** Karlo Toljan, Farren B. S. Briggs

**Affiliations:** 1 Mellen Center for Multiple Sclerosis Treatment and Research, Neurological Institute, Cleveland Clinic, Cleveland, OH 44195 USA; 2 Department of Public Health Sciences, University of Miami Miller School of Medicine, Miami, FL 33136 USA

**Keywords:** Erectile dysfunction, Family planning, Male infertility, Multiple sclerosis, Reproductive health, Sexual dysfunction

## Abstract

**Background:**

Multiple sclerosis (MS) is a chronic neuroinflammatory disease with highest incidence during the period of optimal reproductive health. This scoping review aimed to identify and summarize available data on sexual/reproductive health in males with MS (MwMS).

**Methods:**

This review was based on PRISMA extension for Scoping Review. PubMed database was searched for keyword “multiple sclerosis” alongside keywords “sexual health”, “reproductive health”, “family planning”, “male fertility”, “male infertility”, “sexual dysfunction”, and “erectile dysfunction”, iteratively using the “AND” logical operator. Descriptive analysis was performed on the included articles.

**Results:**

Thirty-four studies were included, and four topics emerged: sexual dysfunction, erectile dysfunction, fertility, and family planning. Sexual dysfunction is common in MwMS (35–72%), yet only a minority of MwMS discuss their sexual health with their treatment teams. Both MS disability and depression were associated with sexual dysfunction in MwMS, with erectile dysfunction and decreased libido as the most prevalent aspects of sexual dysfunction. Positively, phosphodiesterase-5 inhibitors appear effective for treating erectile dysfunction and improving sexual quality of life in MwMS. There may also be a relationship between MS and male infertility, though changes in sexual behavior may underlie this association. Finally, a prominent knowledge gap was observed for disease-modifying therapy use and family planning in MwMS.

**Conclusion:**

Sexual dysfunction is common, impacted by MS severity, and associates with decreased quality of life in MwMS. Communication barriers regarding sexual and reproductive health appear to exist between MwMS and providers, as do literature gaps related to MS therapeutics and sexual/reproductive health.

**Supplementary Information:**

The online version contains supplementary material available at 10.1007/s00415-024-12250-2.

## Introduction

Multiple sclerosis (MS) is a chronic neuroinflammatory disease that adversely affects physical and mental health and can lead to social dissatisfaction [1, 2]. There are 2.8 million people living with MS worldwide [3]. Newer high-efficacy disease-modifying therapies (DMTs) are shifting approaches in care, as risks for relapses substantially decrease with their use [4, 5]. Despite advances in managing disease activity, disease progression persists and many symptoms are difficult to control and worsen over time [6]. In addition to motor impairment in MS, other frequent symptoms include cognitive dysfunction, fatigue, bladder dysfunction, and sleep disturbance, while depression, anxiety, hypertension, and dyslipidemia are common comorbidities [7, 8]. Comprehensive MS care entails management of active symptoms and comorbidities, but there are recognizable challenges due to knowledge gaps for understudied symptoms and lesser recognized comorbidities [9].

### Background

Sexual and reproductive health is severely affected by MS [10–12]. Causes of sexual dysfunction are multifactorial and may be a consequence of neurologic dysfunction, inflammation, hormonal imbalances, and/or due to cognitive dysfunction and mood disorders, which are prevalent in MS [10]. Clinical approaches in MS define primary sexual dysfunction as related to reproductive system dysfunction, secondary as related to disability due to motor impairment, fatigue, or associated bladder and bowel dysfunction, and tertiary as related to psychological and social factors [13]. Sexual dysfunction and impaired reproductive health lead to increased burden of disease, with direct impact on physical health, mental health, and quality of life in MS [12, 14, 15]. Despite the recognized value of sexual and reproductive health in MS, data on these domains are sparse, mostly based on cross-sectional or retrospective studies, and from periods before the availability of newer high-efficacy DMTs [14–22]. Newer DMTs such as B-cell-depleting drugs have different safety profiles than predecessors, and their teratogenic potential is mostly unknown or investigated with ongoing monitoring [21]. In addition, the longitudinal effects of common MS comorbidities on sexual and reproductive health in MS populations remain severely understudied.

The incidence of MS is greatest between the ages of 20 and 40 years [23], which coincides with the age period of optimal fertility, and, thus, family planning should be a part of standard MS care [24]. Studies suggest female infertility may be more common in MS, but causes are not well understood, and recent findings are conflicting [17–19]. Robust data on infertility in males with MS (MwMS) are lacking, but fewer pregnancies in women with MwMS partners compared to the general population have been reported [18, 25]. Similar to females with MS (FwMS), sexual dysfunction is very common in MwMS, with 50% experiencing ejaculatory or orgasmic dysfunction and 40–75% experiencing erectile dysfunction [26–30]. Timely use of effective DMTs is increasingly encouraged to maintain remission, despite known and unknown teratogenic potential [19–22]. Most FwMS discontinue DMT as part of pregnancy planning, though trends over the last decade show an increase in continued use [19]. There are knowledge gaps in MS care regarding the effects of DMT use on sexual and reproductive health in MwMS.

### Clinical relevance and objectives

Following recent reviews focused on female reproductive and sexual health in MS [20, 24, 31], we conducted a scoping review to evaluate the available literature on male reproductive and sexual health in MS. Such a comprehensive summary will facilitate current knowledge synthesis and identify existing gaps in the literature, which will guide next steps in enhancing MS clinical care and research related to male sexual and reproductive health in MS.

## Methods

### Study design and sources

**Fig. 1:**
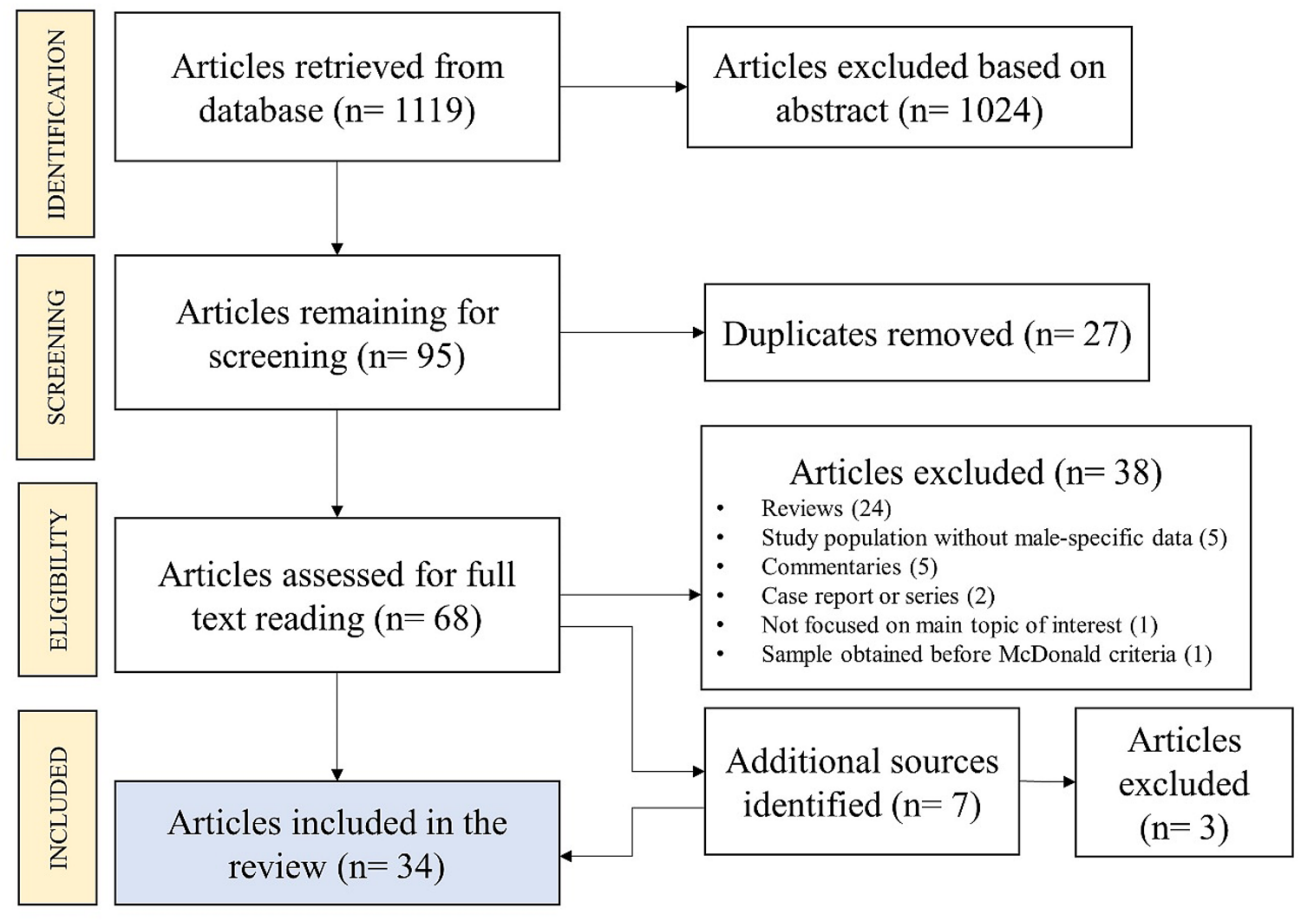
Scoping review flow diagram

To address the stated objective, we conducted a focused scoping review using the PRISMA-ScR Checklist as a methodological guideline [32, 33]. Candidate publications were identified in PubMed database with the use of keyword “multiple sclerosis” and selected keywords (“sexual health”, “reproductive health”, “family planning”, “male fertility”, “male infertility”, “sexual dysfunction”, “erectile dysfunction”), as respective combinations with the logical operator “AND”. Following the initial title and abstract identification, full papers were read for those passing the screening. Secondary sources, which were otherwise not detected with the earlier search, were sporadically identified in retrieved papers and additionally considered for inclusion in the final review based on their relevance. Final included sources had to meet eligibility criteria and were classified by topic, study design, year of publishing, number of male participants, and study population country and continent of recruitment. Specific data on DMTs were not part of this review. Search was performed between July 15th, 2023 and July 24th, 2023. The methodological flowchart with results is presented as Fig. 1.

### Eligibility criteria

Studies available via PubMed database published in English between January 1st, 2002, and July 1st, 2023 were included in the initial screening. The initial date was chosen as a time point after the International Panel on MS Diagnosis presented the recognized diagnostic standard in 2001, which notably integrated magnetic resonance imaging and enabled greater diagnostic accuracy for MS [34]. The final date was chosen as the beginning of the month closest to the time when the literature search was performed. The defined period, spanning more than 2 decades, was also deemed appropriate to assess the overall trends in publishing regarding topics investigated. To broaden the assessed literature, the initial screening included studies regardless of methodology. The final bibliography for the scoping review was manually compiled of original investigations with a focus on MwMS. Duplicates were excluded, as well as reviews, case reports or case series, in vitro or animal studies, and studies that did not report male-stratified findings. The senior author reviewed and approved the final bibliography.

### Synthesis of results

Descriptive analysis was performed for the articles included in the scoping review. Main results are presented in table form and discussed.

## Results

Initial search based on keyword combinations identified 1119 articles, of which 1024 were excluded based on title and abstract review (Fig. 1). Of the 95 articles remaining for screening, 27 duplicates were excluded, and 68 and were assessed for full reading. Further, 37 articles were excluded based on eligibility criteria. From the eligible articles, seven additional sources were identified, and three of those were excluded due to not fully meeting eligibility criteria. The final scoping review included 34 articles.

### Summary of study characteristics

**Fig. 2:**
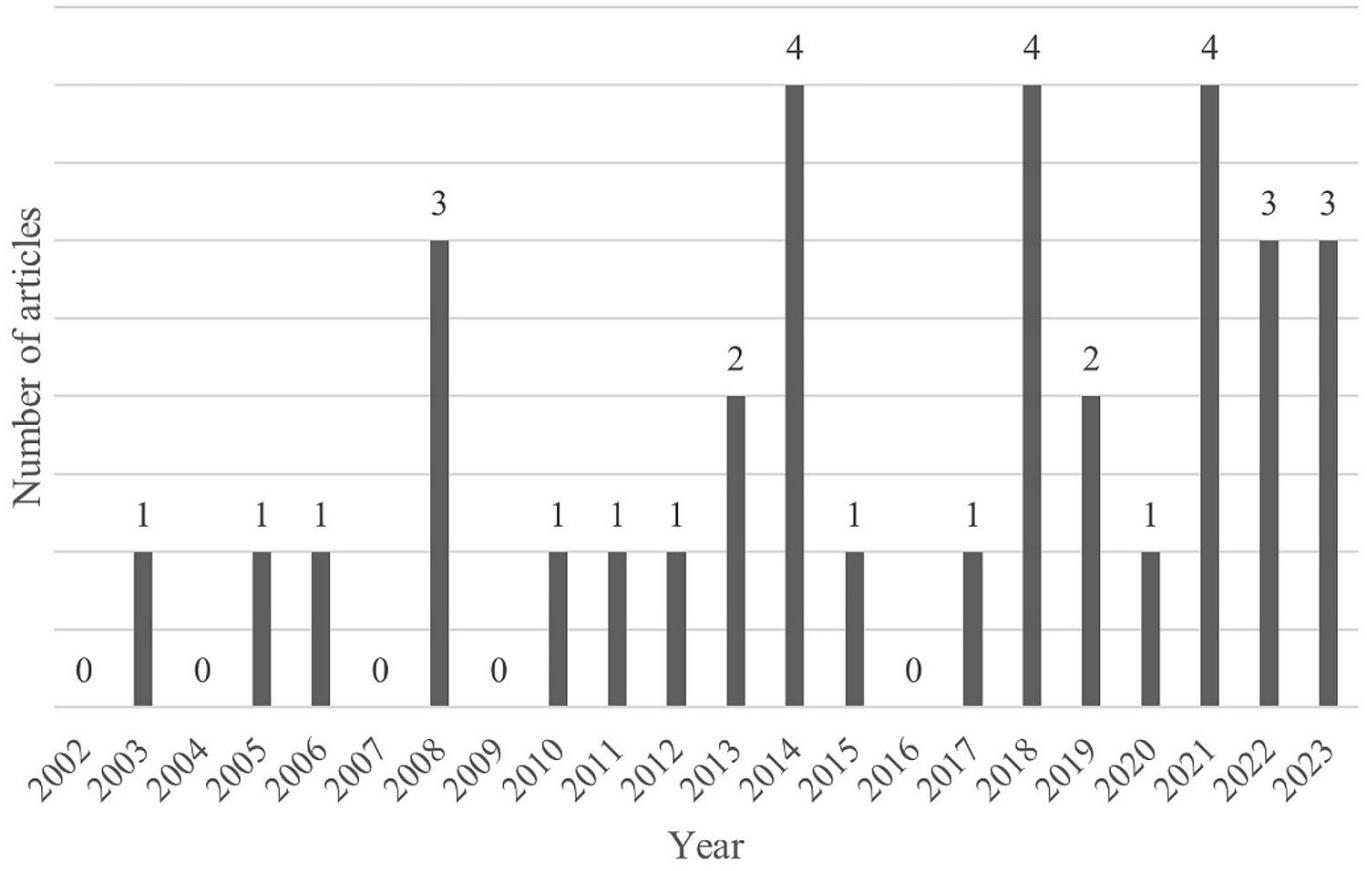
Number of published articles per year and those included in the final scoping review

Information on study types, topics of studies, and geographical locations where studies were conducted is displayed in the Supplemental Table 1. Four main study topics emerged following final review: sexual dysfunction covering multiple domains of sexual health, erectile dysfunction as a specific topic of interest, fertility, and family planning. Most studies were cross-sectional (21/34, 62%) and from Europe (22/34, 65%). Sexual dysfunction in a broader sense was the most frequently addressed topic (20/34, 58%), followed by studies focused on erectile dysfunction (7/34, 21%), fertility (5/34, 15%), and family planning (2/34, 6%). Cohort studies (*N* = 9) were more common than clinical trials (*N* = 2) or case–control studies (*N* = 2). Of the two clinical trials, both investigated treatment for erectile dysfunction. Three studies were international: a clinical trial, a prospective cohort, and a cross-sectional study. Temporal trend showed that half of the included studies (17/34, 50%) were published after 2017 (Fig. 2), with at least one publication every year since 2017. Conversely, there were years between 2002 and 2017 which did not yield any studies for the final scoping review literature (Fig. 2).

### Sexual dysfunction

A summary of studies focused on sexual dysfunction is provided in Table 1. Cross-sectional studies were the most used methodology (16/20, 80%), and three studies were based on cohorts (3 prospective and 1 retrospective). More than half of the studies were European (12/20, 60%) and a fifth were North American (4/20, 20%). The largest study was based on North American Research Committee on Multiple (NARCOMS) Registry and included 1568 male participants [29], while the smallest included 12 male participants [35]. Most studies used standardized questionnaires such as MS Quality of Life [26, 30, 36–38], MS Intimacy and Sexuality Questionnaire [29, 35, 39–46], Sexual Quality of Life Questionnaire for men [35, 44, 47, 48], and International Index of Erectile Function [44, 47, 48], but other methods also included original surveys created by the investigators [46, 49], or other validated forms [38, 39, 46, 49–51]. Serum laboratory tests [42] and magnetic resonance imaging (MRI) [35] were uncommon across studies. Based on instruments applied, sexual dysfunction was predominantly determined as a composite qualitative outcome comprising a sexual quality of life metric in combination with measurements of erectile dysfunction and issues with libido, orgasm, or ejaculation. Across studies, the reported prevalence of sexual dysfunction was 35–72% (Table 1). Common associated factors were depression [36, 38], older age [36, 38, 44, 47], and disability due to MS [36, 38, 43, 44, 50]. Additional factors which were reported as associated with sexual dysfunction in individual studies were fatigue [30, 36] and smoking [44]. A cohort study including 27 MwMS determined a continued decrease in sexual activity and worsening sexual function over a 6-year period [49]. The study also reported that those affected were more willing to discuss sexual dysfunction with their partners than with their treatment team (33% vs 7%) [49], but a later cross-sectional study noted that the presence of family or friends during a clinical encounter can be a barrier to help seeking for sexual dysfunction [46]. In the same study, 6/20 MwMS reported that other MS symptoms overshadow their sexual problems, and 5/20 felt there was insufficient time to discuss sexual function during the encounter [46]. In a cross-sectional study of 50 MwMS investigating serum sex hormone profiles including 17-beta estradiol, progesterone, androstenedione, dehydroepiandrosterone-sulfate, total testosterone, estrone, prolactin, sex hormone-binding globulin, inhibin B, and anti-Mullerian hormone, there were no substantial differences in those with or without sexual dysfunction, except for lower levels of inhibin B in those with sexual dysfunction [42]. No specific brain or spinal cord MRI findings were found to be associated with the severity of sexual dysfunction [35].

**Table 1:** Overview of studies focused on sexual dysfunction in MS, based on male populations, or mixed populations in which a male subgroup was defined

Authors, year, country	Study design	*N*	Methods	Main findings for male population studied
Kaplan et al., 2023, United States [36]	Retrospective cohort	176	MSQoL-54 Modified Fatigue Impact Scale Center for Epidemiologic Studies Depression Scale Descriptive analysis, comparison, correlations, and linear regression (adjusted)	Low sexual function baseline prevalence was 32% and low sexual satisfaction baseline prevalence was 45% Older age, depression, and greater disability due to MS were associated with lower sexual satisfaction Fatigue was associated with sexual dysfunction
Di Pauli et al., 2023, Austria [42]	Cross-sectional	50	MSISQ-19 Multiple Sclerosis Impact Scale-29 Serum sex hormone levels Descriptive analysis and comparison	Sexual dysfunction prevalence was 40% Hormonal profiles were mostly similar in those with and without sexual dysfunction, except lower inhibin B levels in those with sexual dysfunction
de Melo et al. 2023, Brazil [40]	Cross-sectional	92	MSISQ-19 Descriptive analysis and comparison	One third felt their body is less attractive (32%), worried about sexually satisfying their partner (34%), and reported erectile dysfunction (33%) A quarter felt less confident about their sexuality due to MS and feared being sexually rejected due to MS (24%)
Seyman et al., 2022, Canada [35]	Cross-sectional	12	MSISQ-19 SQoL-M Brain and spinal cord MRI Descriptive analysis, comparison, and linear regression (adjusted)	Most subjects had moderate impact of MS on their sexual quality of life There was no independent association of sexual dysfunction severity and quantitative brain and spinal cord MRI metrics when controlling for mood and fatigue
Sabanagic-Hajric et al., 2022, Bosnia and Herzegovina [37]	Cross-sectional	45	MSQoL-54 Descriptive analysis and comparison	More than half (60%) reported the presence of sexual dysfunction
Altmann et al., 2021, Austria [26]	Cross-sectional	40	MSISQ-19 MSQoL-54 Descriptive analysis, comparison, and logistic regression (adjusted)	Sexual dysfunction was commonly reported (45%)
Nabavi et al., 2021, Iran [44]	Cross-sectional	320	Male Sexual Health Questionnaire SQoL-M IIEF-15 MSISQ-19 General Health Questionnaire Descriptive analysis and logistic regression (adjusted)	More than a third reported sexual dysfunction (36%) Factors associated with sexual dysfunction were age, greater disability due to MS, smoking, and worse mental health
Wu et al., 2020, International [30]	Prospective cohort	367	MSQoL-54 Descriptive analysis, comparison, and logistic regression (adjusted)	More than half reported lack of sexual interest (53%) and erectile dysfunction (58%) Perceived cognitive impairment was associated with sexual dysfunction, even when adjusted for age, disability severity, disease duration, marital status, fatigue, depression, comorbidities, and physical activity
Pasic et al., 2019, Croatia [45]	Cross-sectional	26	MSISQ-15 Descriptive analysis and comparison	A third reported erectile dysfunction (35%), and almost a quarter worried about sexually satisfying their partner (23%), felt less confident about sexuality (23%), less masculine (23%), and less attractive (23%) due to MS
Tudor et al., 2018, United Kingdom [46]	Cross-sectional	20	MSISQ-15 ASEX Original 29-item survey Original 23-item Survey Descriptive analysis and comparison	Most reported erectile dysfunction (70%), and 40% reported feeling less confident about their sexuality due to MS Presence of family or friends was seen as a common barrier to help-seeking for sexual dysfunction (40%), as well as other MS symptoms overshadowing sexual problems (30%)
Kisic-Tepavcevic et al., 2015, Serbia [49]	Prospective cohort	27	Original 16-item survey Szasz sexual functioning scale Descriptive analysis, comparison, and generalized linear latent and mixed models (adjusted)	Over 6-year follow-up, sexual activity declined (4% vs. 19% sexually inactive), as well as libido (48% vs 70%), while inability to ejaculate increased (25% vs 52%) A third were willing to discuss sexual dysfunction with partners (33%), but less with a treating provider or friend (7%)
Lew-Starowicz, Rola, 2014, Poland [47]	Cross-sectional	67	SQoL-M IIEF-15 Descriptive analysis, comparison, and correlations	Most subjects had erectile dysfunction (52.9%) and 43% were not satisfied with their overall sexual life SQoL-M scores correlated with most IIEF-15 domain scores A minority discussed their sexual concerns with providers (6%)
Lew-Starowicz, Rola, 2014, Poland [48]	Cross-sectional	67	SQoL-M IIEF-15 Beck Depression Inventory Descriptive analysis, comparison, and correlations	Greater symptoms of depression were associated with decreased sexual function tests scores Older age was associated with lower scores for sexual desire
Fragalà et al., 2014, Italy [43]	Cross-sectional	60	IIEF-15 MSISQ-19 Descriptive analysis, comparison, and logistic regression (adjusted)	Greater disability due to MS was associated with greater erectile dysfunction, but not with overall sexual dysfunction
Orasanu et al., 2013, United States [29]	Cross-sectional	1,568	MSISQ-19 Descriptive analysis and comparison	Erectile dysfunction was common (41%), as well as issues with achieving an orgasm (36%) A third reported decreased libido (30%), and a quarter worried about sexually satisfying their partner (26%)
Celik et al., 2013, Turkey [39]	Cross-sectional	45	MSISQ-19 ASEX Descriptive analysis and comparison	Sexual dysfunction was common (49%) Frequently reported symptoms included erectile dysfunction (20%), decreased confidence about sexuality (17%), lack of libido (13%), and anorgasmia (13%)
Tepavcevic et al., 2008, Serbia [38]	Cross-sectional	31	MSQoL-54 Szasz Sexual Functioning Scale Hamilton Depression Rating Scale Hamilton Anxiety Rating Scale Descriptive analysis and comparison	More than half reported reduced libido (55%) and erectile dysfunction (52%) Problems with ejaculation were common (45%) Age, disability due to MS, being retired, depression, anxiety, and fatigue were associated with sexual dysfunction
Fraser et al., 2008, United States [50]	Cross-sectional	32	Guy’s Neurological Disability Scale Descriptive analysis and correlations	Most subjects reported sexual dysfunction (59%), including issues with erection or ejaculation (28%), or problems which completely prevented sexual activities (16%) Sexual dysfunction was associated with lower limb disability and bladder dysfunction There was no association between sexual dysfunction and age or years since MS diagnosis
Demirkiran et al., 2006, Turkey [41]	Cross-sectional	18	MSISQ-19 Descriptive analysis and comparison	Most subjects reported erectile dysfunction (72%), decreased libido (64%), and anorgasmia (53%)
McCabe et al., 2003, Australia [51]	Prospective cohort	120	Index of Sexual Satisfaction Sexual Dysfunction Scale Sexual Function Scale WHOQoL-100 Descriptive analysis and comparison	Erectile dysfunction was more common with MS than in general population (37% vs 11%), as were problems with ejaculation (29% v s 11%) Having no sexual dysfunction was common in general population (43%), but not with MS (16%)

*ASEX* Arizona Sexual Experience Scale, *IIEF-5(-15)* International Index of Erectile Function-5(-15), *MRI* magnetic resonance imaging, *MS* multiple sclerosis, *MSISQ-15(-19)* Multiple Sclerosis Intimacy and Sexuality Questionnaire-15(-19), *MSQoL-54* Multiple Sclerosis Quality of Life-54, *N* number of male subjects in the study, *SQoL-M* Sexual Quality of Life Questionnaire for men, *WHOQoL-100* World Health Organization Quality of Life-100

### Erectile dysfunction

A summary of studies focused on erectile dysfunction is provided in Table 2. More than half of the included studies were cross-sectional (4/7, 57%), two were clinical trials, and one was a case–control study. Besides the case–control study based on a national database (38,139 cases with erectile dysfunction and 262,848 controls) [52], the second largest study was a randomized double-blind placebo-controlled clinical trial including 217 participants [53]. All studies included the International Index of Erectile Function questionnaire as the main assessment method, with the addition of quality of life [27, 53–55] or urinary tract function [27, 54, 56] metrics in some. In cross-sectional studies assessing the prevalence of erectile dysfunction in MwMS, the values were 45% [28] and 74% [27]. Depression, urinary tract symptoms, and greater disability due to MS were factors associated with erectile dysfunction in MS [27, 54, 56]. Diagnosis of MS was shown to be associated with erectile dysfunction in the large Taiwanese case–control study based on their national insurance database [52]. Phosphodiesterase-5 inhibitors (sildenafil, tadalafil), were shown to be effective for erectile dysfunction in MS leading to improvement in sexual quality of life in two clinical trials [53, 55]. In an international multi-center randomized controlled trial assessing sildenafil (104 subjects) against placebo (113 subjects), after 3 months, 90% of those using sildenafil (25–100 mg dose) reported improvement in erectile function and quality of life in comparison to 24% in the placebo group, a result which was sustained in the 48-week open label extension [53]. In an Italian single-arm prospective study assessing tadalafil, 70 of 92 participants noted improvement in erectile function and quality of life as measured at 3 months [55]. Both trials supported phosphodiesterase-5 inhibitors as safe pharmacological interventions with caveats regarding exclusion criteria, notably uncontrolled cardiovascular comorbidities and major psychiatric disorders [53, 55].

**Table 2:** Overview of studies focused on erectile dysfunction in MS, based on male populations, or mixed populations in which a male subgroup was defined

Erectile dysfunction				
Authors, year, country	Study design	*N*	Methods	Main findings
Bientinesi et al., 2022, Italy [54]	Cross-sectional	57	Dyadic Adjustment Scale ICIQ-MLTUS IIEF-5 Descriptive and linear regression (adjusted)	Urinary tract dysfunction, ED, and greater disability due to MS were associated with negative effects for partnered relationships
Tomé et al., 2019, Brazil [56]	Cross-sectional	41	IIEF-5 ICSmSF Descriptive analysis and comparison	Greater disability due to MS and lower urinary tract dysfunction were associated with ED
Balsamo et al., 2017, Italy [27]	Cross-sectional	101	IIEF-15 SQoL-M International Prostate Symptom Score Beck Depression Inventory Descriptive analysis and logistic regression (adjusted)	Most subjects reported ED (74%) Depression and prostate symptoms were associated with ED
Keller et al. 2012, Taiwan [52]	Case–control	38,139 cases with ED (262,848 controls)	IIEF-5 based on a national health insurance registry Logistic regression (adjusted)	After adjustment for age, urbanization level, and index time of ED diagnosis, those with ED were 2.4 times more likely to have been diagnosed with MS than those without ED Additional adjustment for monthly income, geographic location, vascular comorbidities, and alcohol use still supported the same association (odds ratio 2.2)
Lombardi et al., 2010, Italy [55]	Clinical trial	96	IIEF-15 Sexual Encounter Profile Questions 2 and 3 Life Satisfaction Checklist Descriptive analysis and comparison	After 12 weeks of pre-intercourse tadalafil use (10 mg or 20 mg dose), most subjects (72.9%) experienced improvement in ED and sexual quality of life
Dachille et al., 2008, Italy [28]	Cross-sectional	124	IIEF-15 Descriptive analysis	A third reported moderate or severe ED (29%), and 16% had mild ED 38 subjects (30%) started sildenafil (50 mg or 100 mg) to treat ED
Fowler et al., 2005, International [53]	Clinical trial	217	IIEF-15 Global Efficacy Questions Life Satisfaction Checklist Descriptive analysis and comparison	Subjects who took sildenafil (25- 100 mg) had a greater improvement in ED (90%) versus the placebo group (24%), which was also reflected in improved sexual quality of life

*ED* erectile dysfunction, *ICIQ-MLTUS* International Consultation on Incontinence Questionnaire Male Lower Urinary Tract Symptoms Module, *ICSmSF* International Continence Society for males, *IIEF-5(-15)* International Index of Erectile Function-5 (-15), *MS* multiple sclerosis, *N* number of male subjects in the study, *SQoL-M* Sexual Quality of Life Questionnaire for men

### Fertility

Four cohort studies (3 retrospective and 1 prospective) and a case–control study were focused on fertility as the main topic (Table 3). The prospective cohort study was the smallest (32 participants), but included longitudinal serum sex hormone profiles and sperm analysis [57]. It demonstrated no changes in measured hormonal or sperm parameters over a 12-month follow-up period in those treated with natalizumab or ocrelizumab. The larger retrospective study [58], based on the pooled data from Danish national registries for infertility and multiple sclerosis (24,011 with male-factor infertility, 49 MwMS), showed that male infertility was associated with a presence of diagnosis of MS (odds ratio 1.6), but not with subsequent new diagnosis of MS. In a similar manner, a Swedish case–control study (497 MwMS and 1081 controls) showed there was an association between MS diagnosis in men and being childless for the 5 years preceding index MS clinical symptom with an odds ratio 0.6 for a diagnosis of MS for those with children when compared to being childless [18]. A prior Danish national retrospective cohort based on a sample of 2,240,000 men (3426 MwMS) showed a reduced risk of MS diagnosis in men who had a child, with more children further decreasing the risk of MS diagnosis [25]. However, these case–control studies might reflect changes in sexual behaviors that might lead to conception in the prodromal period for MS. Finally, in a cohort treated with mitoxantrone (238 participants, 80 MwMS), there were no differences in number of pregnancies or rates of abortion or miscarriages between FwMS and partners of MwMS [59].

**Table 3:** Overview of studies focused on fertility and family planning in MS, based on male populations, or mixed populations in which a male subgroup was defined

Authors, year, country	Study design	*N*	Methods	Main findings
Fertility				
D’Amico et al., 2021, Italy [57]	Prospective cohort	32	Serum sex hormone levels Sperm analysis Descriptive analysis and comparison	Measured hormonal and sperm parameters were similar regardless of MS diagnosis When compared to baseline, after 12 months of disease-modifying therapies (natalizumab or ocrelizumab), there were no changes in measured hormonal or sperm parameters in MS group
Glazer et al., 2018, Denmark [58]	Retrospective cohort	51,063	Cross-sectional and survival analysis based on national registries for fertility treatment and MS Logistic and Cox regression (crude and confounder adjusted)	Based on a national registry of couples who underwent fertility treatment, male factor infertility was associated with a diagnosis of MS (odds ratio 1.61), but not with a subsequent new diagnosis of MS
Frau et al., 2018, Italy [59]	Retrospective cohort	80	Original survey Descriptive analysis and comparison	There were no differences in number of pregnancies and rates of abortion or miscarriage between women with MS and partners of men with MS, in the period before or after treatment with mitoxantrone
Hedström et al., 2014, Sweden [18]	Case–control	497 cases with MS (1,081 controls)	Original questionnaire regarding reproductive history Logistic regression analysis (adjusted)	There was an association between MS diagnosis and not having a child in the 5 years prior to index MS symptom (odds ratio for MS was 0.6 for those with children vs childless)
Nielsen et al., 2011, Denmark [25]	Retrospective cohort	2,240,000	National Danish Civil Registration system, respectively, cross-matched with Danish Birth, Hospital, and MS Registers Log-linear Poisson regression analysis (adjusted)	Having children was associated with a decreased risk of MS diagnosis (relative risk 0.89), with number of children being inversely associated with risk of MS diagnosis (in the case of being a male parent of 4 children, relative risk was 0.74)
Family planning				
Bonavita et al., 2021, International [62]	Cross-sectional	61	Original survey Descriptive analysis	Most of the subjects reported that MS did not have an impact on their plans of having children (49%), a quarter significantly changed their plans, and 8% decided against having children due to MS diagnosis
Rasmussen et al., 2018, Denmark [61]	Cross-sectional	102	Original survey Descriptive analysis	Information about fetal risks with disease-modifying therapies was commonly obtained from MS treatment team (40%) organization websites (27%), or social media (13%) Majority did not know if their current disease-modifying therapy had direct teratogenic risks (74%), or if disease-modifying therapies of male partners with MS may be associated with future teratogenic risks for the female partner without MS (85%)

*MS* multiple sclerosis, *N* number of male subjects in the study

### Family planning

Two cross-sectional studies focused on family planning [60, 61] and both were based on original surveys. The first, based on 102 MwMS in Denmark, reported information about fetal risk with use of DMT was commonly obtained from MS treatment team (40% of respondents), but a majority of participants did not know if their current DMT had direct teratogenic effects (74%), or if DMT of male partners with MS may be associated with teratogenicity in case of conception with a female partner without MS [61]. In the second study, in an international sample of 61 MwMS, 49% reported their MS diagnosis did not have an impact on their desire to have children, 8% decided not to have children due to their diagnosis, and 25% reported changing their plans significantly [62].

## Discussion

Our scoping review based on male sexual and reproductive health in MS included 34 original studies published over the last 2 decades (2002–2023). Most studies broadly focused on sexual dysfunction [26, 29, 30, 35–51], followed by specific focus on erectile dysfunction [27, 28, 52–56], fertility [18, 25, 57–59], and family planning [61, 62], respectively. Despite the growing availability of DMT options in recent years, including newer high-efficacy treatments, comorbidity management which includes sexual and reproductive health, remains one of the cornerstones to improve quality of life and minimize direct or indirect disability due to MS [63].

### Multiple sclerosis and male sexual health

Prevalence of sexual dysfunction in global male populations increases with age, especially after the age of 40 years and even more after age of 70 years, but a majority (> 50%) still retain sexual desire [64]. Erectile dysfunction affects 20% of otherwise healthy 50-year-old men, but the prevalence doubles in those with hypertension, obesity, and diabetes [65]. In contrast to a general male population, a much greater proportion of MwMS are affected with erectile dysfunction or loss of libido (40–75%) [26–30]. In the Taiwanese case–control study, the association between erectile dysfunction and a diagnosis of MS remained evident even when controlling for age, socio-economic status, and comorbidities [52]. Although erectile function and libido are the most commonly considered factors associated with male sexual health, etiology of sexual dysfunction in MS is complex, and postulated factors have been clinically organized into a tripartite hierarchical model [13]. For MwMS, our summary identified erectile dysfunction as the most common factor categorized as a component of primary sexual dysfunction, disability due to MS categorized as secondary, and depression categorized as tertiary. Specific hormonal or neuroimaging findings pointing to sexual dysfunction in MwMS have not been identified, aside from a potential role of lower levels of inhibin B [42]. A prior electrophysiologic study based on volunteer sample of 29 MwMS reported neurogenic causes as more frequent than isolated psychological (26 vs. 3 participants), though the latter was also recognized as a potential co-factor when the former is present [66]. Besides optimizing prevention and management of comorbidities found to impact sexual health in the general male population, our review highlights reduction of the burden of motor disability due to MS and improving mental health as additional intervention targets to ameliorate sexual dysfunction in MwMS. In review of the epidemiologic evidence, there were only two clinical trials focused on erectile dysfunction in MwMS. Most of the observational studies on sexual dysfunction were cross-sectional (14/20), which precluded inferences about longitudinal relationships between associated factors. Despite general study design limitations, including modest sample sizes, similar manifestations and related symptoms or comorbidities were reported across studied populations.

### Multiple sclerosis and male reproductive health

There is a major knowledge gap regarding DMT use in the context of reproductive health in MwMS population [61], which possibly has a direct impact for about a third of MwMS who change their plans regarding having children following an established diagnosis of MS [62]. Sexual health is an integral part of reproductive health, but fertility and fecundity also depend on male factors such as sperm quality. Exact pathophysiologic mechanisms of male-factor infertility in MS remain to be determined, though up to 40% of causes of male-factor infertility are elusive even in general population-based studies [67]. Scandinavian population-based studies showed an association between male diagnosis of MS and fewer offspring, and may be indicative of increased infertility but it may also reflect altered sexual behaviors [18, 25, 58]. Most of the observational studies on these topics were retrospective (4/7) and based on specific European populations (4/7 Scandinavian countries, 2/7 Italy), which limits generalizability.

## Limitations

Limitations of this study are primarily related to the nature of the scoping review methodology and chosen strategy, i.e., we restricted our selection of eligible publications to PubMed database for initial article retrieval and defined a specific period. In case an additional source was sporadically identified in one of the read articles, it was considered for inclusion based on same eligibility criteria. Although this enabled an additional number of sources to be included, grey literature on potentially relevant topics and publications in language other than English were not reviewed. With the goal of summarizing the available literature in a broader, yet focused manner, only descriptive reporting or analysis was used. Evaluation of the quality of the included studies was not performed. Aside from research based on national registries, most studies were modestly sized (< 100 participants) and therefore potential subject to sampling variability and from which limited inference is possible.

## Conclusions and future directions

Literature on sexual and reproductive health in MS is predominantly based on female populations, but there may be a trend of a growing scientific interest for male populations for similar aspects of health. Sexual dysfunction in a broader sense has emerged as the topic with most included studies, and our literature review showed a greater geographical diversity for those studies starting in 2020, with otherwise prior dominance of European data. Sexual dysfunction is more prevalent in MwMS than in the general male population, and it is associated with worse quality of life, depression, and disability due to MS. This is potentiated by communication barriers for disclosing sexual dysfunction. Phosphodiesterase-5 inhibitors may improve erectile dysfunction, a very common manifestation of sexual dysfunction. There is scarce data on family planning in MS from male perspective. Additional epidemiological and clinical efforts are needed to further investigate the apparent association of male infertility and MS diagnosis. For better understanding of reproductive health in MS, larger and geographically more diverse studies are needed in male populations, ideally based on prospective registries.

### Supplementary Information

Below is the link to the electronic supplementary material.

Supplementary file1 (DOCX 20 kb)
