# Supplementary material for: Male sexual and reproductive health in multiple sclerosis: a scoping review
Source: J Neurol. 2024 Feb 28;271(5):2169–81. doi: 10.1007/s00415-024-12250-2 (PMC11055735; doi:10.1007/s00415-024-12250-2)
Supplement: Supplementary file 1 — Supplementary file1 (DOCX 20 kb) [file 415_2024_12250_MOESM1_ESM.docx]

Supplemental table 1. Characteristics of articles included in the scoping review (n=34).

| Study type (n) | Topics addressed (n) | Continent and country (n) |
| --- | --- | --- |
| - Cross-sectional (21) - Prospective cohort (5) - Retrospective cohort (4) - Clinical trial (2) - Case-control (2) | - Sexual dysfunction (20) - Erectile dysfunction (7) - Fertility (5) - Family planning (2) | - Europe (22)   - Italy (7)   - Denmark (3)   - Austria (2)   - Poland (2)   - Serbia (2)   - Turkey (2)   - Bosnia and Herzegovina (1)   - Croatia (1)   - Sweden (1)   - United Kingdom (1) - North America (4)   - United States (3)   - Canada (1) - South America (2)   - Brazil (2) - Australia (1) - Asia (2)   - Iran (1)   - Taiwan (1) - International (3) |
